# Effectiveness of Silanization and Plasma Treatment in the Improvement of Selected Flax Fibers’ Properties

**DOI:** 10.3390/ma14133564

**Published:** 2021-06-25

**Authors:** Weronika Gieparda, Szymon Rojewski, Wanda Różańska

**Affiliations:** Institute of Natural Fibres & Medicinal Plants—National Research Institute, Wojska Polskiego 71B, 60-630 Poznań, Poland; szymon.rojewski@iwnirz.pl (S.R.); wanda.rozanska@iwnirz.pl (W.R.)

**Keywords:** natural fibers, modification, plasma treatment, silanization, flammability, thermal stability, scanning electron microscopy, fibers evaluation

## Abstract

The study investigated the effectiveness of the combination of chemical and physical methods of natural fibers’ modification. The long flax fibers were subjected to various types of modification. These were silanization, plasma modification and a combination of these methods. For the silanization process, two types of silanes were used: amino- and vinylsilane. The application of structurally different compounds allowed us to acquire knowledge about the effect of the modifier structure on its properties. Various properties of flax fibers were investigated, comparing the results before and after different modification processes. The flammability of prepared samples were tested by pyrolysis combustion flow calorimeter (PCFC). In the effect of the natural fibers’ modifications, flammability was reduced even by 30%. The thermal stability of modified fibers increased. The FTIR tests of the gases released during thermal degradation of the tested fibers allowed us to determine the important compounds and prove a lower degree of flax-fiber decomposition after modification. Flax fibers were also tested to evaluate their physical properties (linear mass, average diameter, aspect ratio and hygroscopicity). Changes in surface morphology were observed by scanning electron microscope (SEM). The properties of natural fibers improved significantly, thus contributing to an increase in their suitability for the use in composites.

## 1. Introduction

Natural fibers are, among others, one of the fillers in the polymers, in order to improve the properties of the finished products. Polymer materials are an integral part of our everyday life. It is important to note that the usage of synthetic materials that are not biodegradable has polluted the environment to an alarming level [1]. Better awareness and willingness to care for the environment makes people more often turn to natural materials. Products manufactured from pure polymers are frequently replaced by those made from composites filled with natural raw materials. Decades ago, only the textile and packaging industries used natural fibers. However, advances made in the field of natural fibers and their hybrids constitue a worthy alternative to materials used in numerous conventional industries, such as the construction, aerospace or automotive industries [2]. Implementing natural-fiber-based materials instead of synthetic composites in a vehicle can result in a significant (up to 40%) reduction of weight. In this industry, many interior items were made exclusively of pure polymers, and this situation created a fire hazard. There can be many solutions to this problem, such as the addition of flame retardants or various types of synthetic, mineral or natural additives. Each approach has certain advantages, but the use of a natural filler in the form of lignocellulosic natural fibers seems to be the most appropriate one. Numerous polymer elements of vehicles can be replaced with bio-composites reinforced with natural fibers, e.g., interior insulation, seat bottom, door panels, dashboard, body panels, boot liner, etc. By their use, not only reduction of flammability of the prepared element, but also increased biodegradability and reduced mass, and thus reduced weight of the whole vehicle, can be obtained, leading to lower fuel consumption. Despite these advantages, composites with natural fibers have a few weaknesses, such as poor chemical and fire resistance, poor melting temperature, poor interfacial bonding between matrix and fibers, poor moisture absorption, etc. [1,3]. The disadvantages mentioned make it necessary to conduct surface treatment of the natural fibers before incorporating them into composites. In order to achieve good parameters, they should be modified also for further flammability reduction. For this purpose, either chemical or physical methods can be used.

Chemical structure of fibers and polymer matrix are different. Natural fibers consist of hemicellulose, lignin, pectin, water and waxy soluble substances [4,5]. In composites, to the hydrophobic polymer matrix, hydrophilic natural fibers are introduced. As a result, ineffective stress transmission across the interface of the composites is observed due to poor adhesion [4,6]. To deal with this problem, chemical methods of natural-fiber modification, such as mercerization, acetylation, benzoylation, steric acid treatment and silanization, can be used [6,7]. The silanization method offers a wide range of possibilities, due to the possibility of using compounds with various chemical structures adapted to the type of polymer matrix used in the composite [8]. This method involves reacting with hydroxyl groups on the surface of the fibers. However, its undoubted advantage is that the silanes not only react with the fibers, but also condense to form a thin protective coating that is able to give additional properties. Numerous studies have shown that the modification of natural fibers by silanization has a positive effect on improving the water resistance of the fibers, increasing the surface wettability of natural fibers by polymers and promoting interfacial adhesion, and additionally reduces flammability and improves their thermal stability [9,10]. As a result, the properties of natural-fiber-reinforced composites are improved, e.g., flammability or mechanical properties such as tensile strength, flexural modulus, percentage elongation and water absorption, etc. [7].

In addition to the chemical methods used for natural fibers modification, there are also physical methods that are clean, dry and free from expensive and environmentally unfriendly chemicals [11]. Initial system costs (i.e., cost of equipment) can be high, but operators are not exposed to unsafe processes and system operating costs are minimal. Additionally, the high utilization costs associated with hazardous processes are eliminated in this case. These methods are mainly based on energy transfer to the surface of fibers to activate cellulose functional groups—hydroxyl groups. The energy causes breaking of chemical bond between hydrogen and oxide, and forming free radicals. The examples of these methods include plasma, corona or UV radiation [12,13,14]. Physical modifications induced by plasma treatment of the fibers surface can improve the compatibility with the polymer matrices [15,16]. Plasma treatment provides an opportunity to remove contaminants and weakly bound layers, enhance wettability by incorporating polar groups on the surface and to form functional groups permitting covalent bonding [17,18]. The improved interfacial bonding in fiber composites results in increased mechanical strength [3].

The influence of various types of physical and chemical modification of natural fibers on their properties has been widely analyzed in the literature. Miedzianowska et al. analyzed in her work [19] properties of silanized lignocellulosic filler and its application in natural rubber biocomposites. The influence of the conducted modifications by three types of silanes on the morphology and structure of straw particles was investigated. The increase in hydrophobicity and thermal stability of natural fibers was confirmed in their research. After the modification, the straw structure was less smooth and more dispersed. Kumarjyoti et al. have been studied the suitability of various chemical treatments to improve the performance of jute fibers filled natural rubber composites [20]. The surface of jute fibers was modified by three different surface treatments, alkali treatment, combined alkali/stearic, acid treatment and combined alkali/silane treatment. Interestingly, alkali/silane treatment was found to be most efficient surface treatment method to develop strong interfacial adhesion between natural rubber matrix and jute fibers. Referring in turn to the physical methods of modification of natural fibers Hamad et al. have investigated and quantified the effect of plasma-surface modification on ramie plant fibers [21]. It can be concluded from that paper that such treatment can be an effective method in modifying the fiber surface. The modification carried out in these studies led to a surface roughness which increases the surface area and leads to better wettability and interaction of the fiber with the matrix. It can be assumed that the application of plasma surface modification will bring enormous benefits in the production of fiber–polymer composites.

In this paper, the combination of chemical and physical methods used for the modification of natural fibers was presented. As a pretreatment, in order to improve the properties of natural fibers, a silanization process was performed. For even better results it was decided to use plasma modification after fibers silanization. The plasma process was applied at the end of technological chain of fibers modifications to provide them with more hydrophobic properties, improve silane condensation and give a better adhesion with the polymer matrix.

## 2. Materials and Methods

### 2.1. Materials

Natural fibers: Osmotically degummed flax fibers prepared by INF&MP—NRI (Flax fibers); reagents for silanization: Acetic acid 80% pure p.a. and ethyl alcohol 96% pure p.a. supplied by Avantor Performance Materials Poland S.A., Gliwice, Poland, 3—(diethylenetriamine) propyltrimethoxysilane (silane VII) and vinyl trimethoxysilane (silane VIII) provided by Unisil Sp. Z o.o., Tarnów, Poland.

### 2.2. Fibers

The degumming process was carried out with an experimental device operating in the periodic mode. In this method, the degumming process was based on usage of physical laws, especially of osmosis phenomenon, which is observed inside fibrous plant stems in contact with water. This method ensured obtaining the odor-free fibers without damage, characterized by light color and higher aspect ratio in comparison with the fibers extracted with the use of other methods, e.g., dew retting.

The laboratory tests on the degumming process were run at the 14 kg batches of flax straw. The process was carried out in the following conditions: water temperature of 30 °C, process time of 72 h and water flow rate of 30 dm^3^/min. During the processs, a C-type UV lamp was used for inhibiting the growth of retting microorganisms, which is a typical occurrence in the warm-water retting method. After osmotic degumming, the process of hydrodynamic rinsing of straw with cold water was applied, and then the excess water was wrung. Next, the straw was dried in at approximately 60 °C, for 48 h.

### 2.3. Modification

#### 2.3.1. Silanization

Two silanes with different structure and properties were used for the study—more polar nitrogen-containing aminosilane and less polar-vinylsilane. The work started with the parameter adjustment for fibers modification method. Due to the susceptibility to the rapid hydrolysis of the silanes, and then polymerization of the compound in aqueous solution, a silanization process was carried out in a mixture of water with ethanol in acidic medium (acetic acid). Parameters of the modification process, such as pH, solution’s concentration and modification time, were adjusted: 5% (*w*/*w*) silane (VII or VIII) solution in C_2_H_5_OH/H_2_O (6/4) (*v*/*v*) with pH = 4.5. Modification was conducted for 1 h, at the room temperature. After that, the fibers were drained and placed in a chamber set at 80 °C. Dry fibers were cured for 10 min at 105 °C. The parameters of the modification process were optimized in order to minimize fibers’ damage, and for obtaining the highest reactivity and achieving the best yield of the silanization process.

#### 2.3.2. Plasma Treatment

Plasma treatment was conducted in a two-stage process. Plasma gas argon was used with flow: 143.3 SCCM for preliminary cleaning of the flax, at pressure inside the plasma chamber: 50 mTr, with discharge power 100 W for 1 min. The main plasma treatment was conducted as the second stage of the process in the same pressure, with use of argon (the flow at 143.3 SCCM) and hexamethyldisiloxane (HMDSO) (the flow at 20 SCCM) for 10 min with discharge power 90 W.

### 2.4. Test Methods

#### 2.4.1. Thermal Stability Tests

Thermogravimetric study (TGA) was performed with a TA Instruments Analyser Q50, TA INSTRUMENTS, New Castle, DE, USA. A tested sample (about 20 mg) was subjected to heating within the temperature range from 30 to 650 °C and heating rate of 15 °C/min in nitrogen atmosphere at constant gas flow rate of 90 mL/min.

#### 2.4.2. Fourier Transform Infrared Spectrometry (FTIR) Analysis

During TGA study the released gases were identified. The tests were performed with a TA Instruments iZ10 model, Thermo Fisher Scientific, Madison, WI, USA. The spectrum of the released gases contained 8 scans per second at a resolution of 4 cm^−1^ within the range from 600 to 4000 cm^−1^.

#### 2.4.3. Flammability Tests

Flammability tests were carried out by pyrolysis combustion flow calorimeter (PCFC) from FTT. Tests were performed according to the standard of ASTM D7309-2007. The heating rate was 1 °C/s. Pyrolysis temperature range was 75–750 °C, and the combustion temperature was 900 °C. The flow was a mixture of O_2_/N_2_ 20/80 cm^3^/min and the sample weight was 3–4 mg. The maximum heat release temperature (T_max_) and maximum heat release rate (HRR_max_) were determined.

#### 2.4.4. Evaluation of Fibers

A method of retting of natural fibers for their use in composites was presented in the paper published in Textile Research Journal in 2017 [22].

Flax fibers after modification were tested and compared with unmodified fibers to evaluate their properties: linear mass (tex), average diameter of divided “bundle” of fibers, aspect ratio and hygroscopity (65%).

According to the Polish Standard PN-EN ISO 1973:2011 the linear mass (tex) of natural fibers was determined. Carried out under ambient conditions measurement of mass of separate bundles, made of 100 fibers cut to the length of 10 mm from flax fibers middle sections, made it possible to determine the average linear mass (tex) of fibers.

The average diameter of the fibers was determined on the basis of the surface area of a technical fibers cross-section. The aspect ratio (s) was determined as the ratio between the length (l) and the diameter (d) of the divided “bundle” of fibers, according to Equation (1):

s = l/d,(1)

The cross-section and longitudinal views were photographed using microscopic test conducted with Hitachi S-3400N scanning electron microscope (SEM), Hitachi High Technologies America, Inc., Minato, Japan. The fibers were sprayed with conductive agent (gold) and the test was performed under high vacuum with 500 magnification, voltage 20 kV and working distance 20 mm.

A common problem is the ability of a textile product to absorb water vapor from the air. For the purposes of the research, the hygroscopicity of 65% was determined in accordance with the standard for evaluation of fibers and textiles. This parameter was expressed as the quotient of the difference between the mass of the sample stored in a desiccator at 100% air humidity and the dry mass of the sample by the dry mass of the sample, expressed as a percentage.

## 3. Results

In order to evaluate the effectiveness of the combination of various methods of fibers modification (silanization and plasma), the modification of the fibers was carried out in various variants. Both the silanization and the plasma modification separately, as well as the combined modifications with those methods were carried out.

### 3.1. Thermal Stability

**Figure 1:**
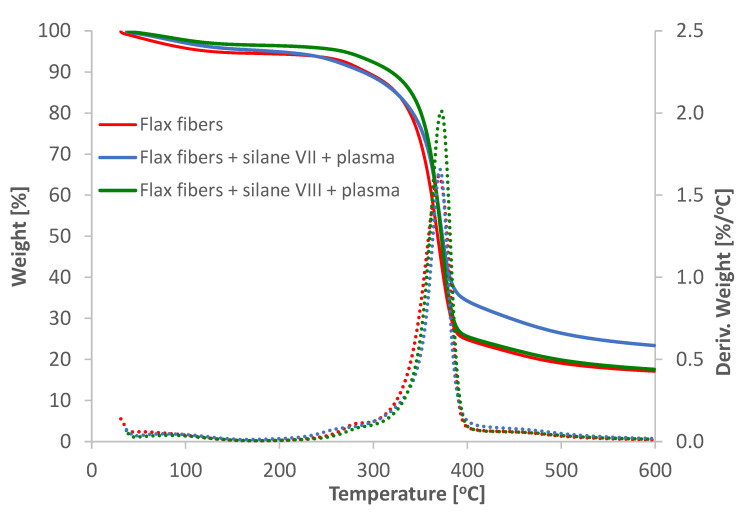
TGA results for unmodified and modified fibers.

The analysis of TGA/DTG curves for untreated flax fibers as well as or flax fibers after two-step modification are shown in the Figure 1. A specific values for the TGA analysis are additionally shown in the Table 1.

The decomposition process can be divided into four stages [23]. First stage, which occurred at the temperature about 100 °C, was water evaporation and was characterized by 3.0–4.6% mass loss. Second stage, in which decomposition was taking place, occurred at the temperature of about 185–300 °C and was characterized by 5.1–12.0% mass loss. Flax fibers that decomposed under these conditions produced mostly carbon dioxide and water. At the third stage the cellulose degradation occurred. It was the main stage of decomposition, mass loss was the highest and reached about 61.3–65.0% at temperature of about 370 °C. Degradation of flax fibers led to production of carbon dioxide, formaldehyde, acetic and formic acids and water. In case of fibers modified by silane VII and plasma, ranging from about 380 °C, decrease of weight loss was observed. This may be due to interactions between conjugated cyclic structures formed from cellulose and the amine groups contained in used silane. “Crosslinking” that occurs between the above mentioned structures may affect the formation of graphite-like structures, which additionally improves thermal stability and increases coal yield [24]. Fourth stage—the longest stage of decomposition, occurred at temperature range from 250 to 600 °C. This stage of decomposition was attributed to slow degradation of lignin and was characterized by 4.6–7.4% mass loss.

It is clear that used treatment of flax fibers improved their thermal stability, as shown by the shifted curves, to higher temperatures compared to untreated fibers. It is visible especially for fibers modified by plasma treatment and vinylsilane (silane VIII). The specific temperatures (T_onset_, T_10_, T_60_) were much higher than those for unmodified fibers as well as DTG peak was slightly shifted to higher temperatures. Numerous studies reported that unmodified fibers had lower decomposition temperature compared to modified fibers [25,26,27,28].

### 3.2. Fourier Transform Infrared Spectrometry (FTIR) Analysis

Compounds such as: carbon monoxide, carbon dioxide, water, acetic acid, formic acid and formaldehyde were determined on the basis of the FTIR analysis of the gases that were released during thermal degradation (TGA) of the fibers. The compounds list was shown in Table 2.

**Figure 2:**
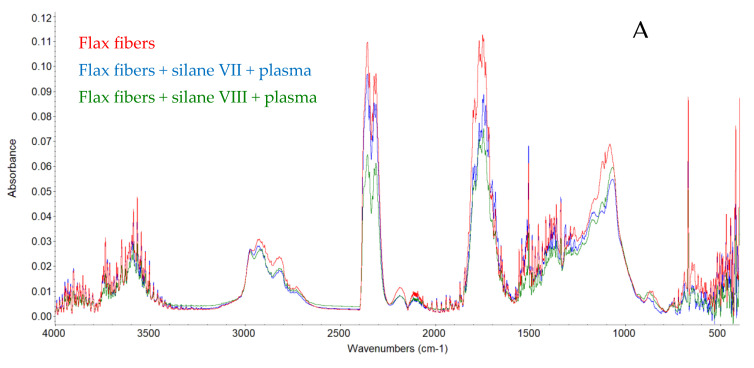

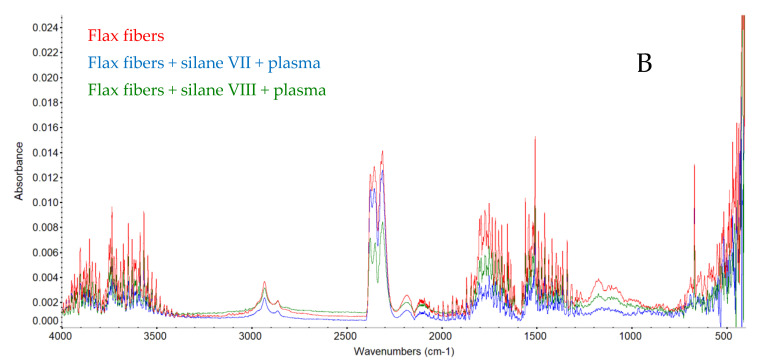
FTIR spectra for III^rd^ step (**A**) and II^nd^ step (**B**) of thermal decomposition unmodified and modified fibers.

FTIR spectra for III^rd^ step (A) and II^nd^ step (B) of thermal decomposition are shown in the Figure 2. Both in the case of the decomposition of the third and second thermal degradation stages, a clear reduction in the peaks responsible for the presence of carbon dioxide, carbon monoxide, acetic acid, formic acid and formaldehyde was observed: CO_2_ at 2355 cm^−1^, C=O at 1770 and 1795 cm^−1^, C-HO at 2780 cm^−1^, and C-O at 1121 and 1177 cm^−1^. This proved a lower degree of flax fibers decomposition and confirmed the assumption that a part of lignin and hemicellulose was removed during the chemical modification process [29].

### 3.3. Flammability

**Figure 3:**
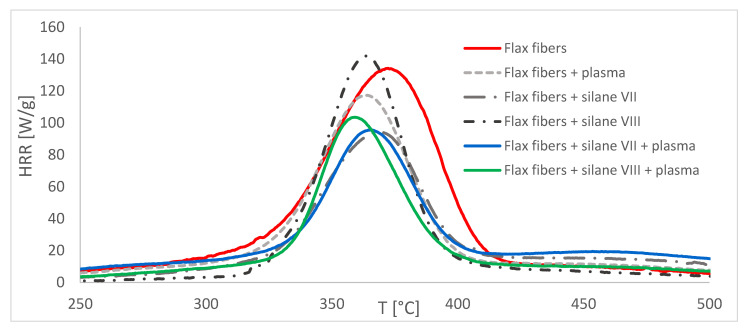
PCFC results for unmodified and modified fibers.

**Figure 4:**
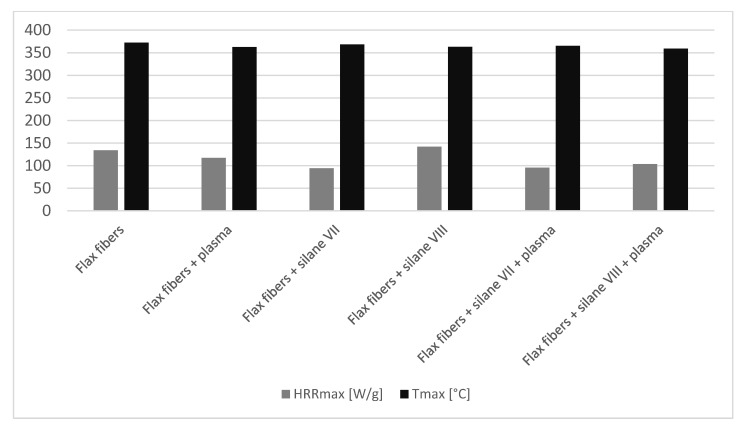
Comparison of HRR_max_ and T_max_ for unmodified and modified fibers.

Combustion parameters of unmodified and modified fibers measured by pyrolysis combustion flow calorimeter (PCFC) are presented in the Figure 3 and Figure 4.

Modification performed only by the silanization method turned out to be effective only in the case of aminosilane (silane VII), which decreased HRR_max_ by 30%. Unfortunately, for vinylsilane (silane VIII), slight increase (6%) of this parameter was observed. Fibers modification with plasma alone led to a 13% decrease in HRR_max_.

The combination of both methods, in the case of the sample after modification with silane VII and plasma treatment, showed no further flammability reduction. HRR_max_ in this case was comparable with the results of the sample modified with silane VII only (approximately 30%). Interestingly, it should be emphasized, that for the sample chemically modified with silane VIII and then physically modified with plasma, there was a reduction of HRR_max_ by 23%. That was a better result than the summary results of both modifications applied separately. Therefore, it is clear that there was a synergistic effect in the combination of the physical and chemical methods of fibers modification. Initial silane coating of the fibers further increased the susceptibility of the fibers to plasma treatment.

For all modifications mentioned in this paper a visible decrease in T_max_ could be observed. This result occured due to the fact that each of the modifications affected the fibers in its own way, changing the structure of its surface by breaking the chemical bonds between hydrogen and oxide, some of the glycosidic bonds, creating free radicals and removing impurities [13,17]. The lowest reduction of T_max_ was observed for fibers modified by silane VII, so the same sample, for which the best result in HRR_max_ was obtained, and it was less than 4 °C. Modification by the same silane VII in combination with plasma resulted in further reduction of T_max_. Interestingly, the biggest reduction of T_max_ was observed for natural fibers modified by silane VIII in combination with plasma. This type of modification resulted in 13° reduction of T_max_,, that was 4 °C more than in case of modification by silane VIII used separately. For sample modified with the use of plasma only, the reduction was almost 10 °C. According to literature, it was also reported that T_max_ can be declined in the modified fibers [28]. Thus, it can be assumed that combining more of them (modifications) together causes a further reduction of this parameter. This may be due to the fact that with each modification fibers of greater purity were obtained.

### 3.4. Evaluation of Fibers

The parameters of untreated fibers were compared with parameters of fibers modified in different process conditions and after each step of modification. Developed chemical and physical surface modification of flax fibers resulted in the changes in main parameters, such linear mass, diameter and aspect ratio, as well as the ability for moisture absorption (Table 3).

Chemical and physical treatment of the fibers caused an increase in their surface area, as well as the structure became rougher. This is helpful, among other aspects, for better interfacial bonding [30]. It was clearly shown that untreated fibers had larger diameters than treated fibers. The chemical modification applied allowed it to attack the fibers’ surfaces and break the lignin and hemicellulose web, and then it separated the fibers from the bundles [29]. The diameter of flax fibers was significantly smaller after silanization and plasma treatment. Importantly, the decreasing diameter of the fibers was also followed by their improved homogeneity. The standard deviation decreased proportionally with decreasing diameter. The treatment with plasma without silanization proved to be most effective treatment in diameter reduction among all the treatments. These results were consistent with those of silanized natural fibers presented in other publications [31]. Another significant change was the increase in the aspect ratio of the fibers. The modification with the plasma itself also had the greatest influence on this parameter. In this case, the aspect ratio increased more than threefold when comparing the degummed fibers and the plasma treated fibers. The most important disadvantage of natural fibers is their hydrophilic nature, which causes a poor interface between the fibers and the matrix in polymer composites. In addition, physical impurities and the presence of hydroxyl groups on the fibers surface make them difficult to use as reinforcing materials [32]. In this study, the hygroscopicity of the fibers was determined both before and after the modification processes. A significant reduction in the hygroscopicity of the fibers was observed, especially for silane-modified fibers in combination with plasma treatment. In this case, the hygroscopicity decreased more than twice. The modification also affected the linear mass (tex) of the fibers, reducing it by 25% compared to the untreated fibers.

Surface morphology of flax fibers before and after silanization, plasma and both were investigated to determine effect of modification processes on the fibers surface morphology. Table 4 shows SEM images of unmodified and modified flax fibers.

The surface condition of fibers is very important with regard to interfacial bonding between the fibers and the polymer matrix for better mechanics properties [33]. From the longitudinal view of flax fibers, it can be observed on the images that there were many impurities on the surface of untreated fibers. After chemical modifications, the surface was cleaner and smoother due to the formation of thin protective layer on it that was constructed from condensed silane. It was observed that the impurities were reduced after the treatment. This is consistent with other silane-modified natural fibers studies [26]. The analysis of SEM images allows for the assumption that silane treatment was a very helpful method for removal of lignin and hemicelluloses from natural fibers. That can enhance interfacial bonding between fibers and polymer [34,35]. In turn, taking into account the cross-section of the fibers, an increase in the specific surface area in the case of modified fibers was visible, as well as significantly changed structure and irregular shape of the fibers in the case of plasma modification was visible. That can be the effect of its excitation by plasma.

## 4. Conclusions

The aim of the study was to evaluate the influence of the combining chemical and physical modification of flax fibers on their properties. Obtained results of the research showed that the performed methodology allowed us to modify fibers successfully. Fibers modified by vinylsilane (silane VIII) and plasma had the best thermal stability among all of the modified fibers. The Fourier transform infrared spectroscopy of released gases during thermogravimetric analysis proved a lower degree of flax-fiber decomposition after modification. Based on the results from PCFC, it can be assumed that, in the case of fibers modification using silanization method with the compounds of suitable construction as pretreatment, followed by the plasma modification, it was possible to obtain a synergistic effect in the flammability reduction. It was shown that both modification using silane and plasma and their combination in a two-step process, with one exception (fibers modified by silane VIII), resulted in a reduction of flammability. SEM results showed no destruction of fibers surface, reduction of impurities on the fibers surface in the case of silane treatment and irregular surface after plasma treatment. The method of the flax fibers’ modification used in this research also had a significant impact on the improvement of such properties of fibers as diameter, specific surface area or their hygroscopicity, which is important in the context of use in composites.

## Figures and Tables

**Table 1:** Values of TGA for long flax before and after modification.

Type of Modification	T_onset_ (°C)	Weight Change (%)	DTG Peak (°C)	Residue at Temperature 600 °C (%)	T_10_ (°C)	T_60_ (°C)
Flax fibers	348.40	65.08	369.49	17.12	292.09	374.55
Flax fibers + silane VII + plasma	354.05	57.69	370.92	23.38	288.73	381.31
Flax fibers + silane VIII + plasma	355.37	66.40	372.14	17.58	318.69	377.34

**Table 2:** The list of detected and identified compounds and their functional groups released during thermal decomposition of flax fibers.

Compound Identified	Molecular Formula	Functional Group	Wave Number cm^−1^
Water	H_2_O	OH	3737
Carbon dioxide	CO_2_	CO_2_	2355; 2311; 671
Carbon monoxide	CO	CO	2182
Acetic Acid	CH_3_COOH	OH C=O C-O -CH_3_	3590 1795; 1770 1177 2976
Formic Acid	CHOOH	OH C=O C-O -CH	3590 1795; 1770 1121; 1067 2910
Formaldehyde	CHOH	C-HO C=O	2810; 2728 1770; 1746

**Table 3:** Evaluation of flax fibers, before and after chemical/physical modification.

Type of Modification	Linear Mass (tex)	SD of Linear Mass (tex)	Averadge Diameter ^1^ (µm)	SD of Diameter (µm)	Aspect Ratio	SD of Aspect Ratio	Hygroscopicity 65 (%)	SD of Hygroscopicity 65 (%)
Flax fibers	0.8	0.04	67.16	38.96	59.56	48.06	7.94	0.32
Flax fibers + silane VII	1.0	0.08	50.15	27.33	103.50	50.30	8.39	0.14
Flax fibers + silane VIII	0.6	0.00	32.68	16.26	146.17	53.11	7.20	0.53
Flax fibers + Plasma	0.6	0.07	24.85	8.93	178.34	53.91	5.39	0.17
Flax fibers + silane VII + Plasma	0.6	0.07	30.50	15.98	158.68	60.64	4.08	0.21
Flax fibers + silane VIII + Plasma	0.7	0.00	33.90	17.81	143.93	55.32	3.18	0.09

^1^ Average diameter of divided “bundle” of fibers.

**Table 4:** SEM of unmodified and modified flax fibers.

Sample	Longitudinal View of Flax Fibers Magnification × 500	Cross-Section View of Flax Fibers Magnification × 500
Flax fibers	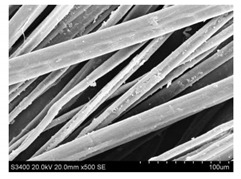	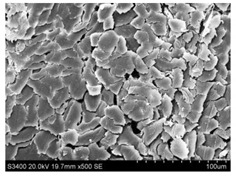
Flax fibers + plasma	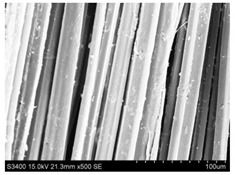	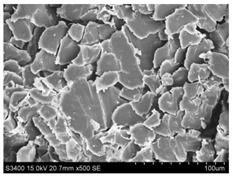
Flax fibers + silane VII	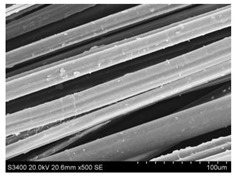	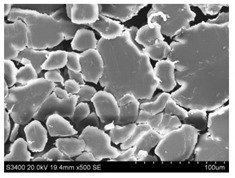
Flax fibers + silane VIII	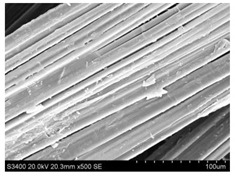	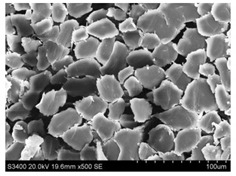
Flax fibers + silane VII + plasma	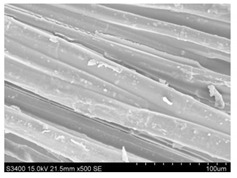	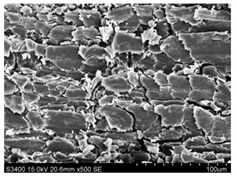
Flax fibers + silane VIII + plasma	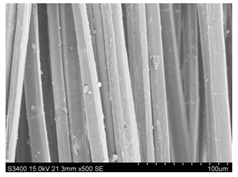	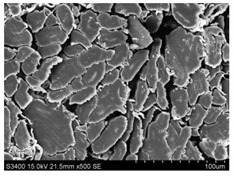

## Data Availability

Not applicable.
